# The impact of hepatic and renal function on panitumumab exposures in patients with metastatic RAS wild-type colorectal cancer

**DOI:** 10.1007/s00280-021-04319-w

**Published:** 2021-07-02

**Authors:** Michael Z. Liao, Hans Prenen, Sandeep Dutta, Vijay V. Upreti

**Affiliations:** 1 Clinical Pharmacology, Modeling and Simulation, Amgen Inc, 1120 Veterans Boulevard, South San Francisco, CA 94080 USA; 2 Antwerp University Hospital, Edegem, Belgium; 3 Clinical Pharmacology, Modeling and Simulation, Amgen Inc, Thousand Oaks, CA USA

**Keywords:** Panitumumab, Pharmacokinetics, Dose adjustment, Hepatic impairment, Renal impairment, *RAS* wild-type

## Abstract

**Purpose:**

Panitumumab is a human monoclonal antibody targeting the epidermal growth factor receptor for the treatment of wild-type *RAS* metastatic colorectal cancer (mCRC). Currently, no dedicated clinical studies have evaluated the effect of organ impairment on the pharmacokinetics of panitumumab. Here, we present data from late phase studies of panitumumab in patients with mCRC and analyses of the effect of hepatic or renal impairment on the exposure of panitumumab.

**Methods:**

From three multicenter, open-label, phase 2 and phase 3 studies, 349 and 351 patients were included in hepatic and renal function subgroup analyses, respectively. Patients who received IV panitumumab and serum exposures were compared to patients with varying degrees of hepatic and renal organ dysfunction.

**Results:**

The C_max_ and C_trough_ values for patients with mild (*n* = 119) and moderate (*n* = 4) hepatic impairment were within the range of serum concentrations of panitumumab for the normal hepatic function subgroup. The distributions of serum concentration of panitumumab in patients with mild (*n* = 85) or moderate (*n* = 19) renal impairment were similar to the serum concentrations of panitumumab in the normal renal function subgroup. Population pharmacokinetic modeling and covariate analysis results were also consistent with lack of any significant effect of renal or hepatic impairment on the pharmacokinetics of panitumumab. Additionally, real-world evidence from case studies of patients with mCRC and severe hepatic or renal impairment, which is a rare patient population to study, indicated lack of clinically relevant differences in exposure of panitumumab compared with patients with mCRC and normal hepatic or renal function.

**Conclusions:**

Mild-to-moderate hepatic or renal dysfunction had no clinically meaningful impact on the pharmacokinetics of panitumumab in patients with mCRC. No dose adjustments for panitumumab are warranted in patients with mCRC with mild-to-moderate hepatic or renal dysfunction.

**Trial registration:**

ClinicalTrials.gov; NCT00083616, NCT00089635, NCT00113763

**Supplementary Information:**

The online version contains supplementary material available at 10.1007/s00280-021-04319-w.

## Introduction

In the United States, colorectal cancer is the third most common cause of cancer death and it is estimated that the number of new diagnoses in 2020 will reach almost 150,000 [1]. Metastasis is present in approximately 20% of patients at diagnosis [1], and patients with metastatic colorectal cancer (mCRC) are likely to present with liver and kidney dysfunction. Monoclonal antibodies (mAbs) targeting the epidermal growth factor receptor (EGFR) have been recommended for treatment of mCRC [2]. EGFR is a transmembrane receptor tyrosine kinase with multiple ligands that promotes cell growth and survival in both normal and malignant cells [3]. EGFR expression has been observed in numerous types of cancer, including gastric, lung, head and neck, ovarian, and bladder carcinomas [4]. Although clearance of mAb therapies occurs primarily through intracellular lysosomal proteolytic degradation, many factors may affect the clearance and exposure of mAbs including neonatal Fc receptor binding, target-mediated drug disposition, and Fc gamma receptor binding [5, 6]. However, pharmacokinetic data for mAbs are limited in patients with hepatic and renal impairment [6, 7].

Panitumumab (Vectibix^®^; Amgen Inc., Thousand Oaks, CA, USA; www.amgen.com) is a human mAb targeting EGFR used for the treatment of wild-type *RAS* mCRC [8, 9]. Panitumumab binds specifically and selectively to the EGFR and prevents the binding of activating ligands (e.g., EGF and transforming growth factor-α). In preclinical studies, the binding of panitumumab to EGFR was demonstrated to reduce EGFR signaling and cause cell cycle arrest [10]. To date, no dedicated phase 1 studies have been conducted for panitumumab in patients with mCRC and hepatic or renal impairment. Here, we present data from three open-label phase 2 and phase 3 studies in patients with mCRC (NCT00083616, NCT00089635, and NCT00113763) to assess the effect of hepatic and renal impairment on exposure to panitumumab. Additionally, these results are comprehensively evaluated together with the limited real-world evidence available for the pharmacokinetics of panitumumab in patients with mCRC and severe hepatic or renal dysfunction, which is a rare patient population to study [11, 12]. The objective of this manuscript was to provide observed panitumumab pharmacokinetics data from mCRC patients with mild-to-moderate hepatic dysfunction and mild-to-moderate renal dysfunction. Here, we also assessed the clinical impact of organ impairment on the pharmacokinetics of panitumumab in patients with mCRC.

## Methods

### Study design and patients

Data for this analysis were pooled from three multicenter, open-label studies: two phase 2 studies (NCT00083616 and NCT00089635 [13]) and one phase 3 study (NCT00113763 [14]). Out of 14 studies in the panitumumab clinical program, these three studies had matching pharmacokinetic, hepatic, and renal data available and were included in this analysis. The two phase 2 single-arm studies enrolled a total of 388 patients and 385 of these patients received panitumumab [13]. The phase 3 study randomly assigned patients 1:1 to receive panitumumab plus best supportive care (231 patients) or best supportive care alone (232 patients) [14]. Patients included in this analysis (≥ 18 years) had confirmed diagnosis of metastatic colorectal carcinoma, Eastern Cooperative Oncology Group (ECOG) performance status ≤ 2, and evidence of disease progression on prior therapies, and had to have pharmacokinetic, body weight, and laboratory results associated with hepatic and renal function available to be included in this analysis. Patients were excluded if they had previous anti-EGFR therapy, previous anti-tumor therapy within 30 days (< 1 week serum half-life) or 3 months (longer serum half-life) before randomization, systemic chemotherapy or radiotherapy within 30 days before randomization, or severe hepatic or renal impairment. Patients received panitumumab administered by intravenous infusion at 6 mg/kg once every 2 weeks. Study protocols were approved by the institutional review boards and independent ethics committees at participating study centers. All patients provided written informed consent before study-related procedures were performed.

### Assessments

Samples for analysis of pharmacokinetics and hepatic and renal function classification were collected at steady state (weeks 7 and 23 of panitumumab treatment). Samples collected at week 23 were not included in this analysis due to missing data. Serum samples were collected 30 min before (trough serum concentration [C_trough_]) and 15 min after (maximum serum concentration [C_max_]) panitumumab administration for measurement of serum concentration of panitumumab using a validated bioanalytical method. A validated bioanalytical (immunoassay with electrochemiluminescence detection) method was used to measure panitumumab concentration in human serum samples. A biotinylated anti-idiotypic antibody to panitumumab was immobilized on streptavidin-coated magnetic beads and was used to capture panitumumab in serum samples [15].

Hepatic function subgroups (normal, mild B1 or B2, moderate, or severe) were defined based on National Institutes of Health (NIH) criteria (Table 1). Liver function was defined using National Cancer Institute Organ Dysfunction Working Group (NCI-ODWG) criteria for hepatic dysfunction, which are based on total bilirubin and aspartate transaminase (AST) [16]. Renal function subgroups were based on creatinine clearance (CL_CR_), which was calculated using actual body weight and the Cockcroft–Gault equation [17]. Renal function was classified as normal (CL_CR_ > 80 mL/min), mildly impaired (CL_CR_ 50–80 mL/min), moderately impaired (CL_CR_ 30–49 mL/min), or severely impaired (CL_CR_ < 30 mL/min).

**Table 1:** Liver function classification

Liver function test	Normal	Mild: B1	Mild: B2	Moderate	Severe
Total bilirubin	≤ ULN	≤ ULN	> 1.0–1.5 × ULN	> 1.5–3 × ULN	> 3–10 × ULN
AST	≤ ULN	> ULN	Any	Any	Any

Liver function classification based on NCI-ODWG criteria for hepatic dysfunction using total bilirubin and AST [16]

*AST* aspartate transaminase, *ULN* upper limit of normal for the institution

Laboratory results were pooled from patients with data available for hepatic and renal function. Descriptive statistics (mean and coefficient of variation [CV]) were used to summarize C_max_ and C_trough_ and were calculated using Phoenix WinNonlin software (version 6.3, Pharsight, St. Louis, MO, USA).

## Results

### Patients

From the three studies, 349 patients were included in the hepatic function subgroup analysis, and 351 patients were included in the renal function subgroup analysis. Patient characteristics are summarized in Table 2. One patient had severe hepatic impairment and was removed from the analysis due to lack of available pharmacokinetic data. No patients had severe renal impairment.

**Table 2:** Demographics and baseline characteristics of patients assigned to panitumumab and used in this analysis (NCT00083616, NCT00089635, and NCT00113763)

	Median	Mean (SD)	*n* (%)
Age			
Male	63.0	61.7 (10.5)	360 (58.2)
Female	59.0	59.3 (10.4)	259 (41.8)
Body weight			
Male	81.8	82.7 (17.0)	360 (58.2)
Female	65.0	69.2 (19.1)	258 (41.7)
Race/ethnicity	–	–	
White	–	–	524 (84.7)
Black	–	–	50 (8.1)
Hispanic	–	–	33 (5.3)
Asian	–	–	7 (1.1)
Other^a^	–	–	5 (0.8)

*SD* standard deviation

^a^Other includes all race categories other than White, Black, Hispanic and Asian

### Hepatic function subgroup analysis

**Fig. 1:**
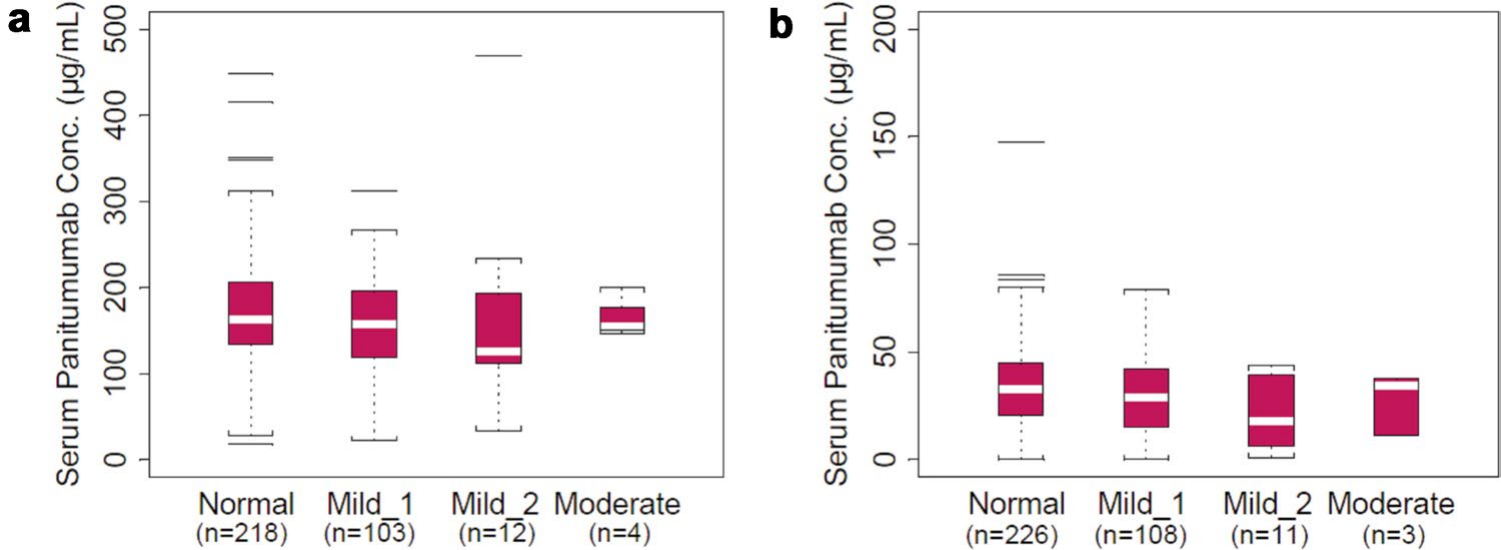
C_max_ (**a**) and C_trough_ (**b**) in patients with varying degrees of hepatic dysfunction on week 7 before and after intravenous infusion of panitumumab at 6 mg/kg every 2 weeks. The "box" in the plot shows the median as a line and the first (25th percentile) and third quartile (75th percentile) of the distribution as the lower and upper parts of the box. The “whiskers” (error bars) above and below the box indicate the 90th and 10th percentiles. The horizontal lines above and below the whiskers are outliers. C_max_, maximum serum concentration; C_trough_, trough serum concentration

Most patients had normal (C_max_, *n* = 218/337, 64.7%; C_trough_, *n* = 226/348, 64.9%), mildly impaired (C_max_, *n* = 115/337, 34.1%; C_trough_, *n* = 119/348, 34.2%), or moderately impaired (C_max_, *n* = 4/337, 1%; C_trough_, *n* = 3/348, 0.8%) hepatic function. Mean (CV) values for C_max_ and C_trough_ were 171 µg/mL (35%) and 34 µg/mL (57%), respectively, for patients with normal hepatic function (Table 3). The distributions of serum concentration of panitumumab were similar among the hepatic function subgroups (Fig. 1). Four patients had moderate hepatic impairment; C_max_ and C_trough_ values for these patients were within the range of serum concentrations of panitumumab for the normal and mild impairment subgroups.

**Table 3:** Summary of serum panitumumab concentrations in patients with varying degrees of hepatic function on week 7 before and after IV infusion of panitumumab at 6 mg/kg Q2W

Liver function	C_max_			C_trough_		
	*n*	Mean, μg/mL	CV, %	*n*	Mean, μg/mL	CV, %
Normal	218	171	35	226	34	57
Mild_1 Impairment	103	158	34	108	30	61
Mild_2 Impairment	12	160	70	11	22	73
Moderate Impairment	4	164	15	3	28	52

*C*_*max*_ maximum serum concentration, *C*_*trough*_ trough serum concentration, *CV* coefficient of variation, *IV* intravenous, *Q2W* every 2 weeks

### Renal function subgroup analysis

**Fig. 2:**
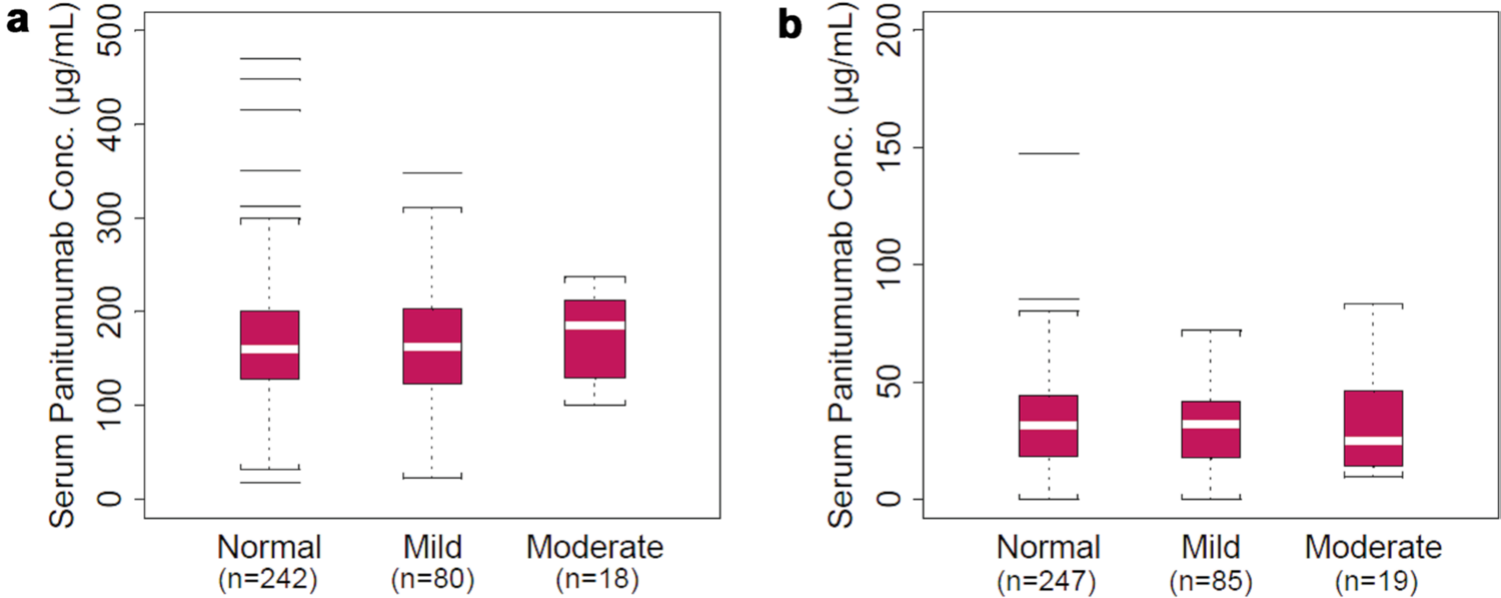
C_max_ (**a**) and C_trough_ (**b**) in patients with varying degrees of renal dysfunction on week 7 before and after intravenous infusion of panitumumab at 6 mg/kg every 2 weeks. The “box” in the plot shows the median as a line and the first (25th percentile) and third quartile (75th percentile) of the distribution as the lower and upper parts of the box. The “whiskers” (error bars) above and below the box indicate the 90th and 10th percentiles. The horizontal lines above and below the whiskers are outliers. C_max_, maximum serum concentration; C_trough_, trough serum concentration

Most patients had normal (C_max_, *n* = 242/340, 71.2%; C_trough_, *n* = 247/351, 70.4%) or mildly impaired (C_max_, *n* = 80/340, 23.5%; C_trough_, *n* = 85/351, 24.2%) renal function; 19 patients had moderate renal impairment (C_max_, *n* = 18/340, 5.3%; C_trough_, *n* = 19/351, 5.4%). Mean (CV) values for C_max_ and C_trough_ were 166 µg/mL (37%) and 32 µg/mL (59%), respectively, for patients with normal renal function (Table 4). The distributions of serum concentration of panitumumab were similar among the three renal function subgroups (Fig. 2).

**Table 4:** Summary of serum panitumumab concentrations in patients with varying degrees of renal function on week 7 before and after IV infusion of panitumumab at 6 mg/kg Q2W

Renal function, CL_CR_ range	C_max_			C_trough_		
	*n*	Mean, μg/mL	CV, %	*n*	Mean, μg/mL	CV, %
Normal, > 80 mL/min	242	166	37	247	32	59
Mild Impairment, 50–80 mL/min	80	165	37	85	30	57
Moderate Impairment, 30–49 mL/min	18	172	27	19	32	68

*CL*_*CR*_ creatinine clearance, *C*_*max*_ maximum serum concentration, *C*_*trough*_ trough serum concentration, *CV* coefficient of variation, *IV* intravenous, *Q2W* every 2 weeks

## Discussion

Panitumumab is indicated for the treatment of mCRC and its population pharmacokinetics have been studied. The pharmacokinetics of panitumumab are best described by a two-compartment population pharmacokinetic model with parallel elimination by a first-order linear pathway and a nonlinear capacity-limited pathway [18, 19]. The linear clearance pathway is a nonspecific proteolytic degradation mechanism mediated by the reticuloendothelial system, similar to other endogenous immunoglobulins. The nonlinear clearance pathway is attributed to panitumumab binding to target EGFR. The panitumumab–EGFR complex is cleared via degradation by lysosomes or cell surface recycling. This pathway is limited by EGFR expression levels and can become saturated when the concentration of panitumumab is increased [19].

There have been no dedicated clinical studies to evaluate the effect of hepatic or renal impairment on the pharmacokinetics of panitumumab in patients with mCRC. The effect of hepatic impairment on the pharmacokinetics of small molecule drugs is typically assessed if hepatic metabolism contributes significantly to the elimination pathway, and liver cytochrome P450 enzymes do not play a role in panitumumab elimination [20]. Nonetheless, the liver has an important role in protein catabolism and could indirectly influence the exposure of panitumumab. Neonatal Fc receptor binding, target-mediated drug disposition, Fc gamma receptor binding, or other elimination mechanisms may be altered in patients with hepatic impairment and could affect the exposure of panitumumab [6]. In this analysis, we demonstrated that mean C_max_ and C_trough_ values of panitumumab for patients with mCRC and mild-to-moderate hepatic impairment were within the range of the mean C_max_ and C_trough_ values for patients with mCRC and normal hepatic function.

**Fig. 3:**
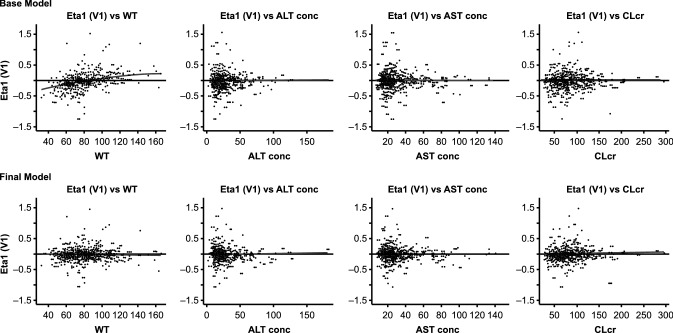
Plot of inter-patient random effect (ETA1) on volume of central compartment (V1) versus covariates (Top Row: base model, Bottom Row: Final model). A reference line at *y* = 0 and local regression smoother trend lines have been included. Panels from the left: ETA1 versus body weight in kg (WT), ETA1 versus ALT concentration in U/L (AST), ETA1 versus AST concentration in U/L (ALT), and ETA1 versus creatinine clearance in mL/min (CL_CR_)

**Fig. 4:**
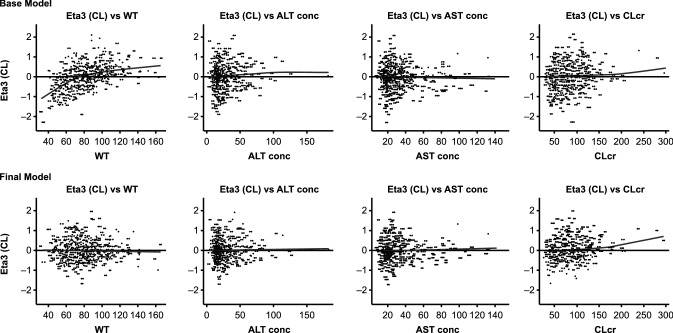
Plot of inter-patient random effect (ETA3) on clearance (CL) versus covariates (Top Row: base model, Bottom Row: final model). A reference line at *y* = 0 and local regression smoother trend lines have been included. Panels from the left: ETA3 versus body weight in kg (WT), ETA3 versus ALT concentration in U/L (AST), ETA3 versus AST concentration in U/L (ALT), and ETA3 versus creatinine clearance in mL/min (CL_CR_)

A population pharmacokinetic analysis was also conducted combining the clinical pharmacokinetic data from the three clinical studies described here along with all the clinical pharmacokinetic data available in the panitumumab clinical development program. The objectives of this additional analysis were to develop a population pharmacokinetic model that describes the disposition of panitumumab in patients with advanced solid tumors and identify the effect of various patient-specific characteristics (covariates) on inter-patient variability in panitumumab pharmacokinetic parameters that may potentially lead to dose adjustments. A two-compartment model with a linear and a Michaelis–Menten elimination pathway adequately described the population pharmacokinetics of panitumumab in patients with advanced solid tumors. Of the available covariates, body weight was found to be the most influential covariate and was able to decrease inter-patient variability in clearance (Fig. 3) and volume of central compartment (V1; Fig. 4). Patient’s hepatic function (AST and ALT) and renal function (CL_CR_) did not correlate with changes in inter-patient variability and were excluded from the pharmacokinetic model (Figs. 3, 4).

Findings from the population pharmacokinetic analysis that demonstrate the lack of organ impairment on panitumumab pharmacokinetics are consistent with the pharmacokinetic results from the three clinical studies and are described here. Among other available covariates, concurrent chemotherapy and tumor type were also able to decrease inter-patient variability in clearance and V1, but to a lesser extent than body weight. All other available covariates, including age and EGFR membrane expression in tumor cells, did not correlate with changes in inter patient variability and were excluded from the pharmacokinetic model. The final model provided precise estimates for all structural pharmacokinetic parameters (relative standard error of the estimate, %RSE < 22%) with inter-patient variability of 53.3 and 24.9% (CV%) for clearance and V1, respectively, with residual variability of 28.8%. The pharmacokinetic parameter estimates (%RSE) of panitumumab V1, clearance, peripheral volume of distribution, inter-compartmental clearance, maximum elimination rate, and Michaelis–Menten constant were 3.22 L (1.32), 0.208 L/day (5.63), 2.49 L (6.14), 0.380 L/day (7.50), 10.1 mg/day (4.47), and 0.501 mcg/mL (21.2), respectively. Simulations indicated that dose adjustments would not be necessary for concurrent chemotherapy or tumor type, as changes in these covariates did not correlate with notable variations in panitumumab exposure. The covariate analysis also showed that, compared with a fixed-dosing scheme, the body weight-adjusted dosing scheme would result in smaller overall inter-patient variability in panitumumab exposure. Hence, body weight is the only patient factor considered in the optimal dosing of panitumumab.

Limited data are available on the effect of renal impairment on the exposure of monoclonal antibodies. FDA guidance recommends conducting a dedicated study to assess exposure of molecules with molecular weight < 69 kDa [21]. A formal pharmacokinetic study of panitumumab has not been conducted in patients with renal impairment; however, an analysis of the effect of renal impairment on exposure of panitumumab is prudent given that patients with renal impairment are likely to receive panitumumab. In this report, mean C_max_ and C_trough_ values for panitumumab for patients with mCRC and mild-to-moderate renal function were within the range of mean C_max_ and C_trough_ values for patients with normal renal function. Glomerular filtration of monoclonal antibodies is limited by the size of the molecule, and glomeruli typically limit compounds with a molecular weight greater than ≈55 kDa [22]. Panitumumab has a molecular weight of 147 kDa [8]; hence, the pharmacokinetics of panitumumab are not meaningfully affected by renal dysfunction. These data are consistent with population pharmacokinetic analyses of panitumumab [18, 19].

Patients with severe hepatic or renal impairment were not included in these studies; thus, we did not assess panitumumab pharmacokinetics in this subgroup of patients. Additionally, there is a paucity of data on the pharmacokinetics of panitumumab in patients with severe organ impairment in the literature. A recent evaluation of therapeutic mAbs highlighted that limited data are available from patients with moderate hepatic impairment (range 0–23 patients per study) and almost no data is available from patients with severe hepatic impairment (range 0–1 patients per study) [6]. Real-world data from a patient with mCRC and severe hepatic impairment receiving panitumumab 6 mg/kg indicated that serum concentrations of panitumumab were not altered compared to patients with adequate liver function [12]. This patient had C_max_ and C_trough_ values of 164 and 10.5 µg/mL, respectively, measured following the second infusion of panitumumab; these values are within the range of serum concentrations of panitumumab reported in our analysis of hepatic function subgroups. Real-world data from a patient with mCRC and chronic kidney disease receiving panitumumab 6 mg/kg [11] showed that serum concentrations of panitumumab were within the ranges presented here for renal function subgroups. The patient’s CL_CR_ was 11 mL/min (severely impaired renal function); C_max_ was 125 µg/mL (after the 11th and 12th infusions) and C_trough_ was 37 µg/mL (just before the 12th infusion). Treatment was well tolerated in both patients and no substantial toxicity was detected. Although these data are limited, no clinically relevant differences in panitumumab exposures were observed compared with available data for patients with normal organ function. Adverse events in the three clinical studies (the two phase 2 studies and one phase 3 study) have been reported [13, 14] and no differences were noted across the normal, mild and moderate organ (hepatic, renal) impaired patients. Panitumumab was overall well tolerated, with the most common adverse events being skin related with skin rash being a well-known characteristic toxicity of EGFR inhibitors. Together, our analysis of patients with mild-to-moderate organ impairment and real-world evidence from patients with severe organ impairment indicate that the pharmacokinetics of panitumumab are not affected by hepatic or renal function. Dose adjustments to the 6 mg/kg once every 2 weeks dosing regimen (recommended in patients with mCRC with normal organ function) are not warranted for patients with hepatic or renal impairment.

## Conclusions

Mild-to-moderate hepatic dysfunction and mild-to-moderate renal dysfunction had no clinically meaningful impact on the pharmacokinetics of panitumumab in patients with mCRC. The 6 mg/kg dose regimen that is recommended in patients with normal organ function is also recommended in patients with hepatic or renal impairment; no dose adjustments are warranted.

## Supplementary Information

Below is the link to the electronic supplementary material.

Supplementary file1 (DOCX 296 kb)

## Data Availability

Qualified researchers may request data from Amgen clinical studies. Complete details are available at the following: http://www.amgen.com/datasharing.
