# Supplementary material for: The impact of hepatic and renal function on panitumumab exposures in patients with metastatic RAS wild-type colorectal cancer
Source: Cancer Chemother Pharmacol. 2021 Jul 2;88(4):665–72. doi: 10.1007/s00280-021-04319-w (PMC8367910; doi:10.1007/s00280-021-04319-w)
Supplement: Supplementary file 1 — Supplementary file1 (DOCX 296 kb) [file 280_2021_4319_MOESM1_ESM.docx]

Vijay V. Upreti, PhD, FCP

Clinical Pharmacology, Modeling & Simulation

Amgen Inc.

1120 Veterans Boulevard

South San Francisco, CA 94080, USA

Email: vupreti@amgen.com

ORCID: 0000-0003-1018-7166

June 16, 2021

RE: Manuscript ID: CCAP-D-21-00062

Dr Etienne Chatelut

Editor-in-Chief

*Cancer Chemotherapy and Pharmacology*

Dear Dr Chatelut:

Thank you for your continued interest in our manuscript titled “The Impact of Hepatic and Renal Function on Panitumumab Exposures in Patients With Metastatic *RAS* Wild-Type Colorectal Cancer” for publication in *Cancer Chemotherapy and Pharmacology.*

We thank you and the reviewer for the comments and suggestions to improve the manuscript. As per the comments, we have conducted population pharmacokinetic analysis of the current data and have summarized it in the revisions to the manuscript. This has further strengthened the manuscript and the analyses. Our manuscript remains within word count, but now contains 4 figures for a total of 8 tables and figures; we trust this is acceptable. Our point-by-point responses and corresponding revisions to the manuscript are detailed in the following pages.

Please do not hesitate to contact me if you have any questions or comments.

Sincerely,

Vijay Upreti, PhD, FCP

**Additional Reviewer Comments:**
The authors did not conduct PopPK and covariate analyses as recommended to address the effect from different covariates. The data are uninterpretable since the demographic characteristics are not matched in arms with different degree of organ impairment. In addition, when subject number is small (e.g., n=3 for Ctrough in moderate hepatic impairment, n=1 for severe hepatic or renal impairment [real-world data]), the conclusion should be inconclusive instead of no effect. Furthermore, AUC was not available for comparison and the authors should using PopPK to compare all relevant PK parameters (e.g., AUC), unless they provide justification that only Cmax and Ctrough correlate with efficacy and safety.

In the reference 1 the authors cited (Population pharmacokinetic analysis of panitumumab in patients with advanced solid tumors. J Clin Pharmacol. 2009;49(10):1142-1156), only the following covariates were analyzed: age, body weight, sex, race, cancer type, concomitant chemotherapy, and EGFR expression (P4). Hepatic and renal impairment were not included in the covariate analyses.

In the reference 2 (Pharmacokinetic and pharmacodynamic perspectives on the clinical drug development of panitumumab. Clin Pharmacokinet. 2010;49(11):729-740), only Cmax and Cmin are compared across arms without covariates analyses (same method as this manuscript).

**Response:** Thank you for your review and comment. We have now undertaken the population pharmacokinetic analysis requested, and have updated the abstract, the introduction, the discussion, and added two additional figures.

***Abstract***

Population pharmacokinetic modeling and covariate analysis results were also consistent with lack of any significant effect of renal or hepatic impairment on the pharmacokinetics of panitumumab. Additionally, real-world evidence from case studies of patients with mCRC and severe hepatic or renal impairment, which is a rare patient population to study, indicated lack of clinically relevant differences in exposure of panitumumab compared with patients with mCRC and normal hepatic or renal function.

***Introduction***

Additionally, these results are comprehensively evaluated together with the limited real-world evidence available for the pharmacokinetics of panitumumab in patients with mCRC and severe hepatic or renal dysfunction, which is a rare patient population to study [11, 12].

***Discussion, paragraph 3***

A population pharmacokinetic analysis was also conducted combining the clinical pharmacokinetic data from the three clinical studies described here along with all the clinical pharmacokinetic data available in the panitumumab clinical development program. The objectives of this additional analysis were to develop a population pharmacokinetic model that describes the disposition of panitumumab in patients with advanced solid tumors and identify the effect of various patient-specific characteristics (covariates) on inter-patient variability in panitumumab pharmacokinetic parameters that may potentially lead to dose adjustments. A two-compartment model with a linear and a Michaelis-Menten elimination pathway adequately described the population pharmacokinetics of panitumumab in patients with advanced solid tumors. Of the available covariates, body weight was found to be most influential covariate and was able to decrease inter-patient variability in clearance (**Figure 3**) and volume of central compartment (V1; **Figure 4**). Patient’s hepatic function (AST and ALT) and renal function (CL_CR_) did not correlate with changes in inter-patient variability and were excluded from the pharmacokinetic model (**Figure 3** and **Figure 4**).

Findings from the population pharmacokinetic analysis that demonstrate the lack of organ impairment on panitumumab pharmacokinetics are consistent with the pharmacokinetic results from the three clinical studies and are described here. Among other available covariates, concurrent chemotherapy and tumor type were also able to decrease inter-patient variability in clearance and V1, but to a lesser extent than body weight. All other available covariates, including age and EGFR membrane expression in tumor cells, did not correlate with changes in inter-patient variability and were excluded from the pharmacokinetic model. The final model provided precise estimates for all structural pharmacokinetic parameters (relative standard error of the estimate, %RSE <22%) with inter-patient variability of 53.3% and 24.9% (CV%) for clearance and V1, respectively, with residual variability of 28.8%. The pharmacokinetic parameter estimates (%RSE) of panitumumab V1, clearance, peripheral volume of distribution, inter-compartmental clearance, maximum elimination rate, and Michaelis-Menten constant were 3.22 L (1.32), 0.208 L/day (5.63), 2.49 L (6.14), 0.380 L/day (7.50), 10.1 mg/day (4.47), and 0.501 mcg/mL (21.2), respectively. Simulations indicated that dose adjustments would not be necessary for concurrent chemotherapy or tumor type, as changes in these covariates did not correlate with notable variations in panitumumab exposure. The covariate analysis also showed that, compared with a fixed-dosing scheme, the body weight–adjusted dosing scheme would result in smaller overall inter-patient variability in panitumumab exposure. Hence, body weight is the only patient factor considered in the optimal dosing of panitumumab.

***Figure 3***

**Fig. 3** Plot of inter-patient random effect (ETA1) on volume of central compartment (V1) versus covariates (Top Row: Base model, Bottom Row: Final model). A reference line at y=0 and local regression smoother trend lines have been included. Panels from the left: ETA1 versus body weight in kg (WT), ETA1 versus ALT concentration in U/L (AST), ETA1 versus AST concentration in U/L (ALT), and ETA1 versus creatinine clearance in mL/min (CL_CR_).

***Figure 4***

**Fig. 4** Plot of inter-patient random effect (ETA3) on clearance (CL) versus covariates (Top Row: Base model, Bottom Row: Final model). A reference line at y=0 and local regression smoother trend lines have been included. Panels from the left: ETA3 versus body weight in kg (WT), ETA3 versus ALT concentration in U/L (AST), ETA3 versus AST concentration in U/L (ALT), and ETA3 versus creatinine clearance in mL/min (CL_CR_).
